# Large-Scale Protein-Protein Interactions Detection by Integrating Big Biosensing Data with Computational Model

**DOI:** 10.1155/2014/598129

**Published:** 2014-08-18

**Authors:** Zhu-Hong You, Shuai Li, Xin Gao, Xin Luo, Zhen Ji

**Affiliations:** ^1^College of Computer Science and Software Engineering, Shenzhen University, Shenzhen, Guangdong 518060, China; ^2^Department of Computing, Hong Kong Polytechnic University, Hong Kong; ^3^Department of Medical Imaging, Suzhou Institute of Biomedical Engineering and Technology, Suzhou, Jiangsu 215163, China

## Abstract

Protein-protein interactions are the basis of biological functions, and studying these interactions on a molecular level is of crucial importance for understanding the functionality of a living cell. During the past decade, biosensors have emerged as an important tool for the high-throughput identification of proteins and their interactions. However, the high-throughput experimental methods for identifying PPIs are both time-consuming and expensive. On the other hand, high-throughput PPI data are often associated with high false-positive and high false-negative rates. Targeting at these problems, we propose a method for PPI detection by integrating biosensor-based PPI data with a novel computational model. This method was developed based on the algorithm of extreme learning machine combined with a novel representation of protein sequence descriptor. When performed on the large-scale human protein interaction dataset, the proposed method achieved 84.8% prediction accuracy with 84.08% sensitivity at the specificity of 85.53%. We conducted more extensive experiments to compare the proposed method with the state-of-the-art techniques, support vector machine. The achieved results demonstrate that our approach is very promising for detecting new PPIs, and it can be a helpful supplement for biosensor-based PPI data detection.

## 1. Introduction

Proteins play crucial roles in cellular biology, including signaling cascades, metabolic cycles, and DNA transcription. In most cases, proteins rarely perform their functions alone; instead, they cooperate with other proteins by forming protein-protein interactions (PPIs) networks. PPIs are responsible for the majority of cellular functions. Over the past decades, many innovative techniques and systems for identifying protein interactions have been developed [1]; for example, in the high-throughput experimental technologies such as yeast two-hybrid (Y2H) screens [2], tandem affinity purification (TAP) [3], mass spectrometric protein complex identification (MS-PCI) [4], and other large-scale biological techniques for PPIs detection, a large amount of PPIs data for different species has been accumulated [5–11]. However, the experimental methods are costly and time consuming; therefore, current PPI pairs obtained from biological experiments only cover a small fraction of the complete PPI networks [12–14]. In addition, large-scale experimental methods usually suffer from high rates of both false positives and false negatives [12, 15–20]. Hence, it is of great practical significance to build low cost protein detection systems and establish the reliable computational methods to facilitate the detection of PPIs [21–25].

A number of computational methods have been proposed for the prediction of PPIs based on different data types, including phylogenetic profiles, gene neighborhood, gene fusion, sequence conservation between interacting proteins, and literature mining knowledge [12, 26–33]. There are also methods that combine interaction information from several different data sources [27]. However, the aforementioned methods cannot be carried out if such biological information about the proteins is not available. Recently, a number of methods which derive information directly from protein sequence are of particular interest [26, 28–30]. Researchers are committed to develop the sequences-based method for discovering new PPIs, and the experimental results showed that the information of amino acid sequences of proteins alone is sufficient to predict PPIs. Among them, one of the excellent works is a support vector machine based method developed by Shen et al. [29]. In that study, the twenty amino acids were firstly clustered into 7 classes according to their volumes and dipoles of the side chains. Then the conjoint triad approach extracts the features of protein pairs based on the classification of amino acids. When applied to predict* human* PPIs, this method yields a high prediction accuracy of about 84%.

Because the conjoint triad approach did not take neighboring effect into account and the interactions usually occur in the discontinuous amino acids segments in the sequence, on the other work Guo et al. developed a method based on SVM and autocovariance to extract the interactions information in the discontinuous amino acids segments in the sequence [26]. Their method yielded a prediction accuracy of 86.55%, when applied to predicting* Saccharomyces cerevisiae* PPIs. Lately, Pan et al. proposed a novel hierarchical LDA-RF model to predict* human* PPIs from protein primary sequences directly. In this study, the local sequential features represented by conjoint triads are firstly extracted from sequences. Then the generative LDA model is used to project the original feature space into the latent semantic space to obtain low dimensional latent topic features. Finally, the random forest model is used to predict the interactions between two proteins. The experimental results show that it is a very promising scheme for PPIs prediction [28].

The general trend in the current study for predicting PPIs has focused on high accuracy but has not considered the running time taken to train the classification model, which should be an important factor of developing a sequence-based method for predicting PPIs because the total number of possible PPIs is very large. For example, if we assume that the* human* genome consists of 22,500 protein-coding genes, then the total number of possible PPIs is estimated to be around 253,113,750 (*N* = 22,500 × (22,500 − 1)/2), which indicates that some classification models with high classification accuracy may not be satisfactory when considering the tradeoff between the classification accuracy and the time for training the models. Here, in addition to exploring the local and global descriptors to mine interaction information from the multiscale amino acids segments at the same time, we also investigate the use of a novel paradigm of learning machine called extreme learning machine (ELM) [34], in order to obtain a balance between high classification accuracy and short training time.

In the present work, we report a novel sequence-based method for the prediction of interacting protein pairs using ELM combined with local and global descriptors. More specifically, we first represent each protein sequence as a vector by utilizing the novel representation of local and global protein sequence descriptors which provides us with a chance to mine interaction information from the multiscale amino acids segments at the same time. Then we characterize a protein pair in different feature vectors by coding the vectors of two proteins in this protein pair. Finally, an ELM model is constructed using these feature vectors of the protein pair as input. To evaluate the performance, the proposed method was applied to* human* PPI dataset. The experiment results show that our method achieved 84.8% prediction accuracy with 84.08% sensitivity at the specificity of 85.53%.

## 2. Materials and Methodology

**Figure 1:**
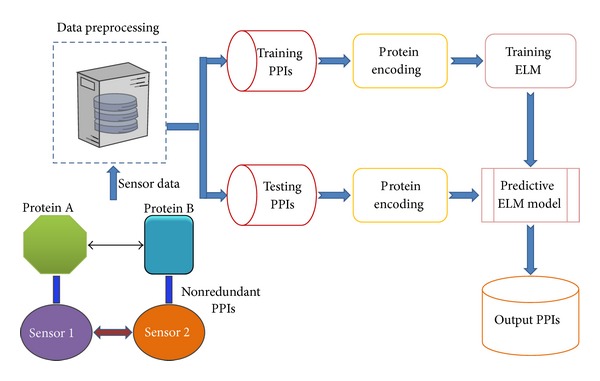
The schematic diagram for mapping large-scale protein-protein interactions by integrating biosensor data with ELM model.

In this section, we outline the main idea behind the proposed method. The flowchart intuitively showing how to map large-scale PPIs by integrating biosensor-based PPI data with computational model is given in Figure 1. Firstly, we discuss the PPI dataset which is used in the study to evaluate the performance of the proposed method. Next we introduce the novel sequence-based protein representation method. Finally, we briefly descript the computational model, ELM, used in this study.

### 2.1. Golden Standard Datasets

We evaluated the proposed method with the* human* PPI dataset, which was downloaded from the Human Protein References Database (HPRD). After self-interactions and duplicate interactions were removed, the remaining 36,630 PPI pairs between 9,630 different human proteins comprise the final positive dataset.

The chosen golden negative dataset has a variable impact on the prediction performance, and it can be artificially inflated by a bias towards dominant samples in the positive data. For golden negative set, we followed the previous work [28] assuming that the proteins in separate subcellular compartments do not interact with each other. In this study, the golden negative dataset is generated from Swiss-Prot database version 57.3 according to four criteria: (1) protein sequences annotated with uncertain subcellular location terms were removed. (2) Protein sequences annotated by multiple locations were removed because of lack of the uniqueness. (3) Protein sequences annotated with “fragment” were removed. (4) Protein sequences with less than 50 amino acid residues were also removed because they might be fragments. After strictly following the above steps, we finally obtained 1,773 human proteins from six subcellular localizations. Then the noninteracting protein pairs were constructed by randomly pairing the proteins from separate subcellular compartments.

We also downloaded the golden negative dataset of human with experimental evidence used in the study of Smialowski et al. [35]. By combining the above two negative datasets, the whole final golden negative dataset consists of 36,480 noninteracting protein pairs. The whole dataset consists of 73,110 protein pairs, where nearly half are from the positive dataset and half are from the negative dataset. Four-fifths of the protein pairs from the positive and negative dataset were, respectively, randomly selected as the training dataset and the remaining one-fifths were used as the testing dataset.

### 2.2. Representing Proteins with Descriptors from Primary Protein Sequences

To successfully use the machine learning methods to identify PPIs from primary protein amino acids sequences, one of the most important computational challenges is how to effectively represent a protein sequence by a fixed length feature vector in which the important information content of proteins is fully encoded [36, 37]. In this study, two kinds of sequence representation approach are used to transform the protein sequences into feature vectors, including amino acid composition and a novel local descriptor. For amino acid composition, it is evident that 20 amino acid composition descriptors reflecting the fraction of each kind of amino acid in a protein sequence are directly calculated. Then, a local multiscale decomposition technique is used to divide protein sequence into multiple sequence segments of varying length to describe local regions. Here, the continuous sequence segments are composed of residues which are local in the polypeptide sequence [38].

In order to extract local information, we first divided the entire protein sequence into seven equal length fractions. Then a novel binary coding scheme was adopted to construct a set of continuous regions on the basis of the above partition. For example, consider a protein sequence “CCYGGGYYCYYYCGGCCYYCG” containing 21 residues. To represent the sequence by a feature vector, let us first divide each protein sequence into multiple regions. For simplicity, the protein sequence is divided into four equal length segments (denoted as S_1_, S_2_, S_3_, and S_4_). Then it is encoded as a sequence of 1's and 0's of 4-bit binary form. In binary format, these combinations are written as* 0000*,* 0001*,* 0010*,* 0011*,* 0100*,* 0101*,* 0110*,* 0111*,* 1000*,* 1001*,* 1010*,* 1011*,* 1100*,* 1101*,* 1110*, and* 1111*. The number of states of a group of bits can be found by the expression 2^*n*^, where *n* is the number of bits. It should be noticed that here 0 or 1 denotes one of the four equal length regions, and S_1_–S_4_ are excluded or included in constructing the continuous regions, respectively. For example, 1100 denotes a continuous region constructed by S_1_ and S_2_ (the first 50% of the sequence). Similarly, 0011 represents a continuous region constructed by S_3_ and S_4_ (the final 50% of the sequence).

It should be noticed that the proposed representation can be simply and conveniently edited at multiple scales, which offers a promising new approach for addressing these difficulties in a simple, unified, and theoretically sound way when presenting a protein sequence. For a given number of bits, each protein sequence may take on only a finite number of continuous or discontinuous regions. This limits the resolution of the sequence. If more bits are used for each protein sequence, then a higher degree of resolution is obtained. In this study, the protein sequence is encoded by 7-bit binary form; each protein sequence may take on 126 (2^7^−2) different regions. Higher bit encoding requires more storage for data and requires more computing resource to process. In this study, only the continuous regions are used and the discontinuous regions are discarded.

For each continuous region, three types of descriptors, composition (*C*), transition (*T*), and distribution (*D*), are used to represent its characteristics. *C* denotes the amino acids number of a particular property (e.g., hydrophobicity) divided by the total amino acids number in a local region. *T* is the percentage frequency with which amino acids for a particular property are followed by protein amino acids of another property. *D* characterizes the chain length within which the first 25 percent, 50 percent, 75 percent, and 100 percent of the protein amino acids of a particular property are located, respectively [39].

The three descriptors can be calculated in the following ways. Firstly, in order to reduce the complexity inherent in the representation of the 20 standard protein amino acids, we firstly clustered them into seven clusters based on the volumes and dipoles of the side chains. Amino acids within the same groups likely involve synonymous mutations because of their similar characteristics [29]. The amino acids belonging to each group are shown in Table 1.

**Figure 2:**
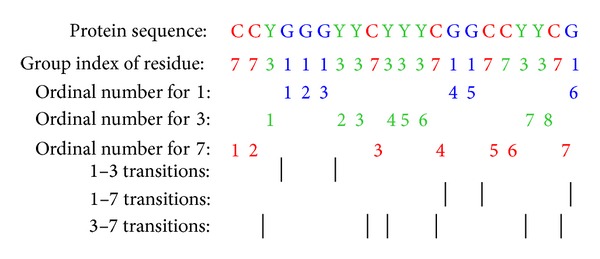
Sequence of a hypothetic protein indicating the construction of composition, transition, and distribution descriptors of a protein region.

Then, every amino acid in each protein sequence is replaced by the index depending on its grouping. For example, protein sequence “CCYGGGYYCYYYCGGCCYYCG” is replaced by 773111337333711773371 based on this classification of amino acids (see Figure 2). There are six “1,” eight “3,” and seven “7” in this protein sequence. The composition for these three symbols is 6/(6 + 7 + 8) × 100% = 28.57%, 8/(6 + 7 + 8) × 100% = 38.10% , and 7/(6 + 7 + 8) × 100% = 33.33%, respectively. There are 2 transitions from “1” to “3” or from “3” to “1” in this sequence, and the percentage frequency of these transitions is (2/20) × 100% = 10%. The transitions from “1” to “7” or from “7” to “1” in this sequence can similarly be calculated as (3/20) × 100% = 15%. The transitions from “3” to “7” or from “7” to “3” in this sequence can also similarly be calculated as (6/20) × 100% = 30%.

**Figure 3:**
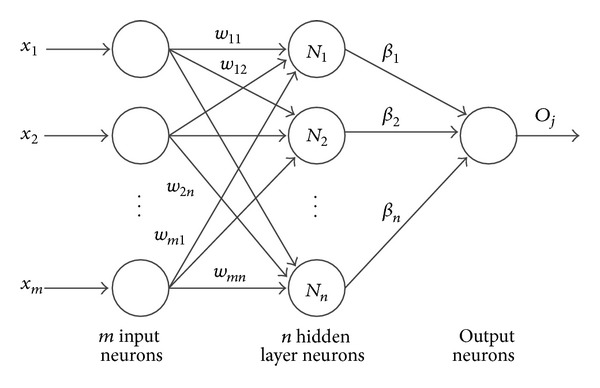
The structure of extreme learning machine.

For distribution *D*, there are 6 residues encoded as “1” in the example of Figure 3, the positions for the first residue “1,” the 2nd residue “1” (25% × 6 = 2), the 4th “1” residue (50% × 6 = 3), the 6th “1” (75% × 6 = 5), and the 8th residue “1” (100% × 6 = 6) in the encoded sequence are 4, 5, 6, 15, and 21, respectively, so the *D* descriptors for “1” are (4/21) × 100% = 19.05%, (5/21) × 100% = 23.81%, (6/21) × 100% = 28.57%, (15/21) × 100% = 71.43%, and (21/21) × 100% = 100%, respectively. Similarly, the *D* descriptor for “3” and “7” is 14.29%, 33.33%, 47.62%, 57.14%, and 90.48% and 4.76%, 9.52%, 61.9%, 76.19%, and 95.24%, respectively.

For each continuous local region, the three descriptors (*C*, *T*, and *D*) were calculated and concatenated, and a total of 63 descriptors are generated: 7 for *C*, 21 ((7 × 6)/2) for *T*, and 35 (7 × 5) for *D*. Then, the local descriptor from 27 regions (7-bit) was concatenated and a total 1701 dimensional vector has been built to represent each protein sequence. Finally, the PPI pair is characterized by concatenating the local and global descriptors of two individual proteins. Thus, a 3442-dimensional vector has been constructed to represent each protein pair and was used as a feature vector for input into SVM classifier.

### 2.3. Extreme Learning Machine

By virtue of their approximation capabilities for nonlinear mappings, the feed-forward neural networks (FNN) have become ideal classifiers in many applications. Huang et al. proved that the single-hidden-layer FNN could exactly learn *M* distinct observations for almost any nonlinear activation function with almost *M* hidden nods [34, 40, 41]. However, the hidden layer biases and input weights of FNN have usually to be tuned using some parameter adjusting approach, which are generally time-consuming due to inappropriate learning steps with significantly large latency to converge to local maxima. Therefore, the slow learning speed of FNN has been a major bottleneck in different applications.

Extreme learning machine (ELM) was originally developed for the single hidden layer feed-forward neural network (SLFNN) and then extended to the generalized SLFNN where the hidden layer need not be neuron alike [34, 40]. As shown in Figure 3, its architecture is similar to that of a SLFNN. Recently the ELM algorithm has been increasingly popular in classification tasks due to its high generalization ability and fast learning speed. Different from the popular thinking that network parameters need to be adjusted, the input weights and first hidden layer biases need not be adjusted but they are randomly assigned in ELM. It has been proved that the ELM algorithm performs learning at an extremely fast speed and achieves a good generalization performance with activation functions which are infinitely differentiable in hidden layers [40, 42, 43].

The ELM algorithm transforms the learning problem into a simple linear system; that is, the output weights of ELM can be analytically determined through a generalized inverse operation of the hidden layer weight matrices. Compared with traditional learning frameworks such a learning scheme can operate at extremely much fast speed. Improved generalization performance of ELM with the smallest training error shows its superior classification capability for real-time applications at an exceptionally fast pace without any learning bottleneck [44].

The basic idea behind ELM algorithm is briefly descripted as follows: suppose learning *N* arbitrary distinct samples (*x*_*i*_, *t*_*i*_) ∈ *R* ^*n*^ × *R* ^*m*^, where *x*_*i*_ = [*x*_*i*1_,*x*_*i*2_,…,*x*_*in*_]^*T*^⊆*R* ^*n*^, *t*_*i*_ = [*t*_*i*1_,*t*_*i*2_,…,*t*_*im*_]^*T*^⊆*R* ^*m*^, a standard ELM with *L* hidden neurons and activation function *g*(*x*) are mathematically modeled by

(1) ∑i=1Lβig(xj)=∑i=1Lβig(wi·xj+bi)=oj, j=1,…,N,

where *w*_*i*_ = [*w*_*i*1_,*w*_*i*2_,…,*w*_*in*_]^*T*^ represents the weight vector connecting the *i*th hidden node and the input nodes, *β*_*i*_ = [*β*_*i*1_,*β*_*i*2_,…,*β*_*im*_]^*T*^ represents the weight vector connecting the *i*th hidden neuron and the output neurons, and *b*_*i*_ is the bias of the *i*th hidden neuron. *w*_*i*_ · *x*_*j*_ denotes the inner product of *w*_*i*_ and *x*_*j*_. A wide variety of functions could be selected as the activation function, including sigmoid function, radial basis function, sine function, hardlim function, and triangular basis function. The architecture of ELM is shown in Figure 3. Equation (1) can be written compactly as

(2) Hβ=T,

where

(3) H(w1,…,wL,b1,…,bL,…,x1,…,xN) =[g(w1·x1+b1)⋯g(wL·x1+bL)⋮⋯⋮g(w1·xN+b1)⋯g(wL·xN+bL)]N×Lβ=[β1T⋮βLT]L×m,  T=[t1T⋮tNT]N×m.

*H* is termed as the hidden layer output matrix of the SLFNN; the *i*th column of *H* is the *i*th hidden neuron's output vector with respect to inputs *x*_1_, *x*_2_,…, *x*_*N*_. Hence for fixed arbitrary input weights *w*_*i*_ and the hidden layer bias *b*_*i*_, training a SLFNN equals finding a least-squares solution $\hat{\beta }$ of the linear system *Hβ* = *T*; that is,

(4) ||H(w1,…,wL,b1,…,bL,x1,…,xN)β^−T|| =min⁡β||H(w1,…,wL,b1,…,bL,x1,…,xN)β−T||.

Equation (12) becomes a linear system and the solution is estimated as

(5) β^=H†T,

where *H* ^†^ is the Moore-Penrose generalized inverse of the hidden layer output matrix *H*.

In summary, given a training dataset *ℵ* = {(*x*_*i*_, *t*_*i*_)∣*x*_*i*_ ∈ *R* ^*n*^, *t*_*i*_ ∈ *R* ^*m*^, *i* = 1,…, *N*}, activation function *g*(*x*), and hidden neuron number *L*, the ELM-based learning procedure can be summarized as follows.

**Step 1 .**

Assign arbitrary input weight *w*_*i*_ and bias *b*_*i*_, *i* = 1,…, *L*.

**Step 2 .**

Calculate the hidden layer output matrix *H*.

**Step 3 .**

According to (13), calculate the output weight *β*.

## 3. Results and Discussion

In this section, we describe our simulation methodology and present the experimental results that evaluate the effectiveness of our schemes. The proposed sequence-based PPI predictor was implemented using MATLAB platform. For ELM algorithm, the implementation by Zhu and Huang available from http://www.ntu.edu.sg/home/egbhuang was used. Regarding SVM, LIBSVM implementation available from http://www.csie.ntu.edu.tw/~cjlin/libsvm/index.html was utilized, which was originally developed by Chang et al. [33]. Tree kinds of kernel functions were chosen and the optimized parameters were obtained with a grid search approach. All the simulations were carried out on a computer with 3.1 GHz 2-core CPU, 8 GB memory, and Windows operating system.

### 3.1. Cross Validation and Performance Evaluation

In the study, fivefold cross-validation technique has been employed to evaluate the performance of the proposed model. In five-fold cross-validation technique, the whole dataset is randomly divided into five subsets, where each subset consists of nearly equal number of interacting and noninteracting protein pairs. Four subsets are used for training and the remaining set for testing. This process is repeated five times so that each subset is used once for testing. The performance of method is average performance of method on five sets.

Seven metrics have been used in the study to measure the predictive ability of the proposed method. The parameters are as follows: (1) the overall prediction accuracy (ACC) is the percentage of correctly identified interacting and noninteracting protein pairs; (2) the sensitivity (SN) is the percentage of correctly identified interacting protein pairs; (3) the specificity (SP) is the percentage of correctly identified noninteracting protein pairs; (4) the positive predictive value (PPV) is the positive prediction value; (5) the negative predictive value (NPV) is the negative prediction value; (6) the *F*-score is a weighted average of the PPV and sensitivity, where an *F*-score reaches its best value at 1 and worst score at 0; (7) Matthew's correlation coefficient (MCC) is a more stringent measure of prediction accuracy accounts for both under- and overpredictions. These parameters are defined as follows:

(6) ACC=TP+TNTP+FP+TN+FN,

(7) SN=TPTP+FN,

(8) $$\begin{array}{c} \text{SP}=\frac{\text{TN}}{\text{TN}+\text{FP}}, \end{array}$$

(9) $$\begin{array}{c} \text{PPV}=\frac{\text{TP}}{\text{TP}+\text{FP}}, \end{array}$$

(10) $$\begin{array}{c} \text{NPV}=\frac{\text{TN}}{\text{TN}+\text{FN}}, \end{array}$$

(11) $$\begin{array}{c} F1=2\times \frac{\text{SN}\times \text{PPV}}{\text{SN}+\text{PPV}}, \end{array}$$

(12) MCC=(TP×TN−FP×FN) ×((TP+FN)×(TN+FP) ×(TP+FP)×(TN+FN))−1/2,

where true positive (TP) is the number of true PPIs that are predicted correctly; false negative (FN) is the number of true PPIs that are predicted to be noninteracting pairs; false positive (FP) is the number of true noninteracting pairs that are predicted to be PPIs, and true negative (TN) is the number of true noninteracting pairs that are predicted correctly.

The above mentioned parameters rely on the selected threshold. The area under the ROC curve (AUC), which is threshold-independent for evaluating the performances, can be easily calculated according to the following formula [45]:

(13) $$\begin{array}{c} \text{AUC}=\frac{S_{0}-n_{0}\left(n_{0}+1\right)/2}{n_{0}\times n_{1}}, \end{array}$$

where *n*_0_ and *n*_1_ denote the number of positive and negative samples, respectively, and *S*_0_ is the sum of the ranks of all positive samples in the list of all samples ranked in increasing order by estimated probabilities belonging to positive. AUC values can give us a good insight into performance comparison of different prediction methods. Although the AUC is threshold-independent, an appropriate threshold must be selected for the final decision. For the classifier which outputs a continuous numeric value to represent the confidence or probability of a sample belonging to the predicted class, adjusting the classification threshold will lead to different confusion matrices which decide different ROC points [29].

### 3.2. Determination of ELM Parameter

The number of hidden nodes is a critical factor for the generalization of ELM. To determine the parameter, four-fifths of the whole dataset are randomly chosen to train the ELM classifiers with different number of hidden nodes, while the rest one-fifths of the dataset are used as the validation set to compute the accuracy.

**Figure 4:**
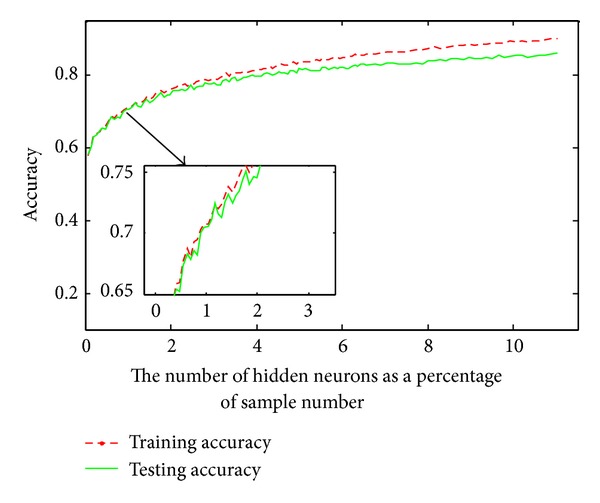
The relationship between the prediction accuracy and the number of hidden neurons. The *x*-axis denotes the number of hidden neurons as a percentage of sample number and the *y*-axis is the corresponding accuracy values.

**Figure 5:**
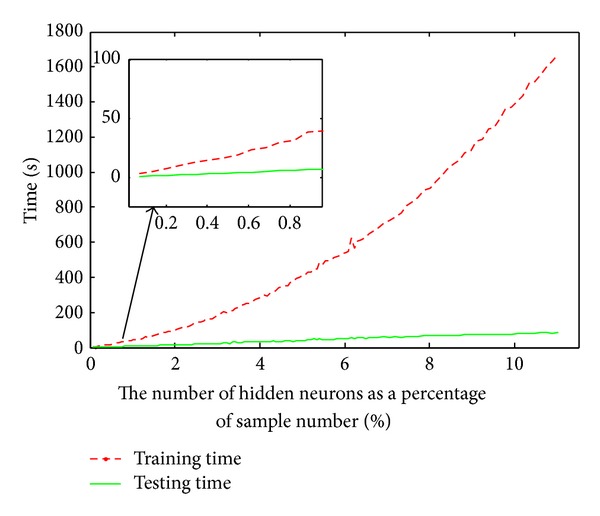
The relationship between the consuming time and the number of hidden neurons. The *x*-axis denotes the number of hidden neurons as a percentage of sample number and the *y*-axis is the running time.

Here the sigmoid function was used as the activation function of the ELM classifier. The results are plotted in Figure 4, which shows that the accuracy value reaches about 0.9 and increases slowly when the number of hidden neurons was set to 9 percent of the amount of samples. Based on Figure 4, we finally set 9 percent of the sample number as the number of hidden neurons for the ELM classifier. The second experiment was to examine how the running time scales with the number of hidden neurons. We increase the number of hidden neurons from 1 to 11 percent of the amount of samples and measure the average time overhead.  Figure 5 shows that the running time of proposed ELM model scales nearly linear as the hidden neuron size increases.

### 3.3. Prediction Performance of Proposed Model

We evaluated the performance of the proposed model using the PPIs dataset as described in the aforementioned section. To guarantee that the experimental results are valid and can be generalized for making predictions regarding new data, we adopted the fivefold cross-validation in this study. The advantages of cross-validation are that the impact of data dependency is minimized and the reliability of the results can be improved.

The prediction performance of ELM predictor with novel representation of protein sequence across five runs is shown in Table 2. It can be observed from Table 2 that high prediction accuracy of 84.8% is achieved for the ELM model with sigmoid function. To better investigate the prediction ability of our model, we also calculated the values of sensitivity, specificity, PPV, NPV, *F*-score, MCC, and AUC. From Table 2, we can see that our model gives good prediction performance with an average sensitivity value of 84.08%, specificity value of 85.53%, PPV value of 85.47%, NPV value of 84.15%, *F*-score value of 84.77%, MCC value of 74.22%, and AUC value of 0.9232. Further, it can also be seen in Table 2 that the standard deviation of accuracy, sensitivity, specificity, PPV, NPV, *F*-score, MCC, and AUC is as low as 0.0022, 0.0019, 0.0028, 0.0040, 0.0038, 0.0029, 0.0030, and 0.0028, respectively.

To demonstrate the performance of the proposed model, we further compared our method with the state-of-the-art predictor SVM. From Table 2, we can see the performance of ELM and SVM model. As observed from Table 2, the testing time of SVM algorithm (2794.29 s) is roughly 38 times the testing time of ELM algorithm (72.7901 s) for sigmoid activation function. In addition, the prediction performance of ELM is also promising. The AUC of the SVM algorithm is 0.8878, which is lower than the ELM. The overall accuracy, sensitivity, specificity, PPV, NPV, *F*1 score, and MCC of SVM algorithm are, respectively, 81.77%, 81.19%, 82.32%, 82.15%, 81.44%, 81.65%, and 70.18% as illustrated in Table 2. Hence, it can be seen that almost all evaluation measures of ELM algorithm are a little better than those of SVM algorithm, while its learning speed is much more faster than SVM.

**Figure 6:**
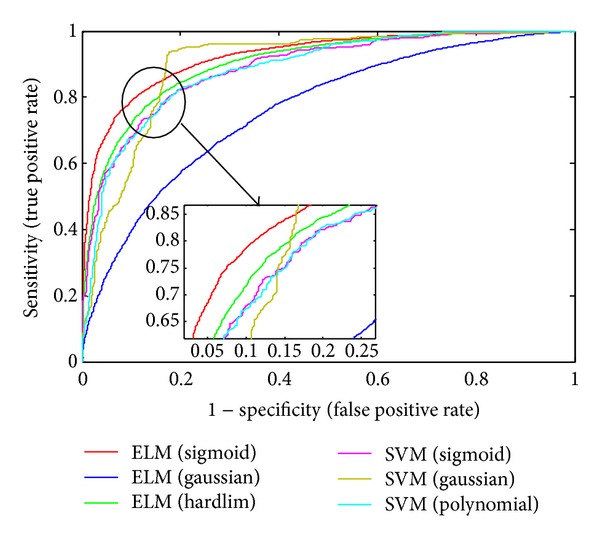
The ROC (receiver operator characteristic) curve illustrating the performance of different activation functions. The curve presents the true positive rate (sensitivity) against the false positive rate (1 − specificity).

We also conduct an experiment to characterize the sensitivity (i.e., the size of true positives that can be detected by our method) and specificity (i.e., 1 − false positive rate) of proposed approach for different activation functions (Figure 6). The results in Figure 6 are reported using receiver operator characteristic (ROC) curves, which plot the achievable sensitivity at a given specificity (1 − false positive rate). Good performance is reflected in curves with a stronger bend towards the upper-left corner of the ROC graph (i.e., high sensitivity is achieved with a low false positive rate). We found that the proposed method achieved over 83 percent detection rate with less than 10 percent false positive rate. The results demonstrate that the proposed ELM can successfully classify positive and negative samples in all five activation functions that we investigated. Our algorithm can perfectly classify interacting and noninteracting protein pairs with only a few exceptions.

To sum up, considering the high efficiency as well as the good performance we can readily conclude that the proposed approach generally outperforms the state-of-the-art model with higher discrimination power for predicting PPIs based on the information of protein sequences. Therefore, we can see clearly that our model is a much more appropriate method for predicting new protein interactions compared with the other methods. Consequently, it makes us be more convinced that the proposed method can be very helpful in assisting the biologist to assist in the design and validation of experimental studies and for the prediction of interaction partners.

## 4. Conclusions

In this paper, we have developed an efficient and fast learning technique, which utilizes global and local information of protein amino acid sequence, for accurate identification PPIs at considerably high speed both in training and testing phase. The first contribution of this work is a novel protein amino acids sequence representation using amino acid composition and a descriptor to represent global and local information of a protein sequence, respectively. Then, the application of extreme learning machine ensures reliable recognition with minimum error and learning speed approximately thousands of times faster than the state-of-the-art classification method SVM. Experimental results demonstrated that the proposed method performed significantly well in distinguishing interacting and noninteracting protein pairs. It was observed that the proposed method achieved the mean classification accuracy of 84.8% using 5-fold cross-validation. Meanwhile, comparative study was conducted on the proposed method and the state-of-the-art SVM. The experimental results showed that our method significantly outperformed SVM in terms of classification accuracy with shorter running time.

## Figures and Tables

**Table 1:** Division of amino acids into seven groups based on the dipoles and volumes of the side chains.

Group	Class	Dipole scale	Volume scale
1	Ala, Gly, Val	Dipole < 1.0	Volume < 50
2	Ile, Leu, Phe, Pro	Dipole < 1.0	Volume > 50
3	Tyr, Met, Thr, Ser	1.0 < dipole < 2.0	Volume > 50
4	His, Asn, Gln, Trp	2.0 < dipole < 3.0	Volume > 50
5	Arg, Lys	Dipole > 3.0	Volume > 50
6	Asp, Glu	Dipole > 3.0 (opposite orientation)	Volume > 50
7	Cys	1.0 < dipole < 2.0 (form disulphide bonds)	Volume > 50

**Table 2:** Comparison of the prediction performance by the proposed method and state-of-the-art SVM classifier on the human dataset.

Method	Kernel	Mean/std	Time (s)	ACC	SN	SP	PPV	NPV	F1	MCC	AUC
			Testing								
ELM	Sigmoid	Mean	72.7901	0.8480	0.8408	0.8553	0.8547	0.8415	0.8477	0.7422	0.9232
		Variance	1.9062	0.0022	0.0019	0.0028	0.0040	0.0038	0.0029	0.0030	0.0028
	Hardlim	Mean	77.4139	0.8206	0.8171	0.8242	0.8227	0.8185	0.8199	0.7056	0.9020
		Variance	3.7710	0.0050	0.0040	0.0063	0.0088	0.0026	0.0063	0.0064	0.0031
	Gaussian	Mean	76.9615	0.7257	0.7328	0.7186	0.7232	0.7283	0.7279	0.6018	0.7624
		Variance	4.1012	0.0036	0.0048	0.0054	0.0085	0.0077	0.0044	0.0033	0.0017

			Training								
ELM	Sigmoid	Mean	1282.12	0.8887	0.8831	0.8944	0.8933	0.8843	0.8882	0.8022	0.9561
		Variance	17.25	0.0006	0.0010	0.0018	0.0014	0.0001	0.0008	0.0010	0.0012
	Hardlim	Mean	1330.33	0.8668	0.8655	0.8682	0.8683	0.8654	0.8669	0.7691	0.9397
		Variance	46.28	0.0027	0.0021	0.0033	0.0027	0.0027	0.0024	0.0039	0.0031
	Gaussian	Mean	1435.45	0.7824	0.7896	0.7753	0.7790	0.7860	0.7843	0.6595	0.8626
		Variance	94.85	0.0033	0.0022	0.0053	0.0040	0.0026	0.0029	0.0037	0.0038

			Testing								
SVM	Sigmoid	Mean	2794.29	0.8177	0.8119	0.8232	0.8215	0.8144	0.8165	0.7018	0.8878
		Variance	16.71	0.0127	0.0266	0.0128	0.0067	0.0200	0.0155	0.0160	0.0143
	Gaussian	Mean	5237.89	0.6947	0.4714	0.9191	0.8535	0.6348	0.6064	0.5320	0.8997
		Variance	67.82	0.0228	0.0412	0.0112	0.0178	0.0265	0.0340	0.0276	0.0364
	Polynomial	Mean	3612.98	0.8019	0.8219	0.7819	0.7903	0.8144	0.8057	0.6820	0.8838
		Variance	20.16	0.0101	0.0126	0.0117	0.0165	0.0114	0.0125	0.0122	0.0138
